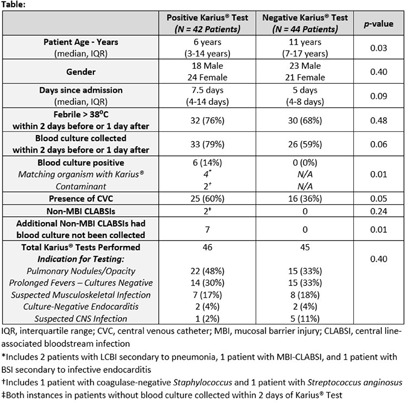# Rethinking the Role of Non-Culture Based Microbiologic Testing Methods within CLABSI Surveillance

**DOI:** 10.1017/ash.2025.294

**Published:** 2025-09-24

**Authors:** Samiksha Tarun, Wendi Beauseau, Sara Griffith, Mindy Bibart, Ethan Mezoff, Amy Leber, Matthew Washam

**Affiliations:** 1 NCH; 2 Nationwide Children’s Hospital; 3 Nationwide Children’s Hospital; 4 Nationwide Children’s Hospital; 5 Nationwide Children’s Hospital

## Abstract

**Introduction:** With the expanding use of non-culture based tests (NCTs), the CDC’s National Healthcare Safety Network (NHSN) incorporated NCTs into the surveillance definition for central line-associated bloodstream infections (CLABSI) in 2016. However, there are limited data available on the impact of NCTs on CLABSI surveillance since that time. In this study, we aim to describe the test performance characteristics of a NCT which detects microbial cell-free DNA (Karius® Test [Redwood City, CA: Karius, Inc]) for bloodstream infection diagnosis and to determine the impact on CLABSI surveillance within a pediatric healthcare facility. **Method:** This study was performed at a 654-bed quaternary care pediatric healthcare facility in central Ohio from January through December 2024. All patients with Karius® Testing performed on or after hospital day 3 were included. Sensitivity, specificity, and positive predictive value (PPV) for Karius® Tests to diagnose bloodstream infections were determined from patients with paired blood cultures collected within 2 days of the Karius® Test. Patients with positive and negative Karius® Tests were compared using Fisher’s exact test and Wilcoxin rank sum test. Analyses were completed using Stata version 18 (College Station, TX: StataCorp LLC). Viral data were excluded from the analysis. Single growth of common commensal organisms on blood culture were treated as contaminants. **Result:** Eighty-six patients with a total of 91 Karius® Test results were included in the analysis (Table; Figure). Patients with a positive Karius® Test were younger and more likely to have a positive blood culture (p Conclusion: In our cohort, the Karius® Test lacked specificity and PPV for the diagnosis of bloodstream infections. This was especially pronounced in afebrile patients in whom clinicians did not suspect bloodstream infection. Inclusion of NCT methods within the CLABSI definition may bias national surveillance rates by including patients with low post-test probability as well as impact diagnostic stewardship efforts to reduce inappropriate blood culture collection.

**Figure:**
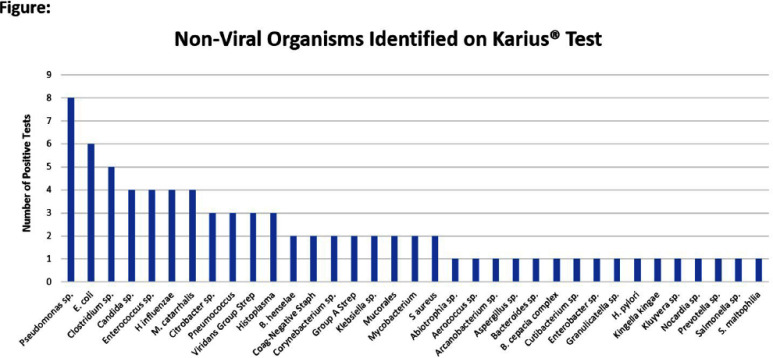

**Figure:**